# Inductively-overcoupled coil design for high resolution magnetic resonance imaging

**DOI:** 10.1186/1475-925X-5-3

**Published:** 2006-01-09

**Authors:** Mehmet Bilgen

**Affiliations:** 1 Hoglund Brain Imaging Center, The University of Kansas Medical School, 3901 Rainbow Blvd, Kansas City, KS 66160, USA; 2 Department of Molecular and Integrative Physiology, The University of Kansas Medical School, 3901 Rainbow Blvd, Kansas City, KS 66160, USA

## Abstract

**Background:**

Maintaining the quality of magnetic resonance images acquired with the current implantable coil technology is challenging in longitudinal studies. To overcome this challenge, the principle of 'inductive overcoupling' is introduced as a method to tune and match a dual coil system. This system consists of an imaging coil built with fixed electrical elements and a matching coil equipped with tuning and matching capabilities. Overcoupling here refers to the condition beyond which the peak of the current in the imaging coil splits.

**Methods:**

The combined coils are coupled inductively to operate like a transformer. Each coil circuit is electrically represented by equivalent lumped-elements. A theoretical analysis is given to identify the frequency response characteristics of the currents in each coil. The predictions from this analysis are translated into experiments and applied to locally image rat spinal cord at 9.4 T using an implantable coil as the imaging coil and an external volume coil as the matching coil.

**Results:**

The theoretical analysis indicated that strong coupling between the coils divides the resonance peaks on the response curves of the currents. Once these newly generated peaks were tuned and matched to the desired frequency and impedance of operation, *in vivo *images were acquired from the rat spinal cord at high quality and high resolution.

**Conclusion:**

After proper implementation, inductive overcoupling provides a unique opportunity for tuning and matching the coil system, and allows reliable and repeatable acquisitions of magnetic resonance data. This feature is likely to be useful in experimental studies, such as those aimed at longitudinally imaging the rat following spinal cord injury.

## Background

Inductively coupled radio frequency (rf) probes have long been used in magnetic resonance imaging (MRI) studies (please see a review on this area in [1], and the references therein). These probes principally consist of two coils (a primary coil (PC) and a secondary coil (SC)) with no physical connection in between. Configurations with a primary matching coil coupled to a secondary volume coil are widely used in traditional applications. Typically, a circuit loop with tuning and matching elements is positioned centrally above the rung of self-resonating low-pass birdcage coil or directly over the window formed by the two rungs and the two end-ring segments of high-pass birdcage coil for mutual coupling [2]. In other arrangements, the SC is configured as a surface coil [3,4], stent coil [5], wireless catheter coil [6] or an implantable coil [4]. In these cases, the resulting combined coil system provides a flexible and versatile environment for locally imaging the underlying tissue at increased signal-to-noise ratio (SNR) and spatial resolution.

We have been successfully using inductively coupled surface and implantable coils in our microimaging studies at 9.4 T [4,7-12]. In our applications, a single-loop SC is tuned to the frequency of interest using fixed capacitive and inductive elements while a single-loop pickup coil is tuned at much higher off-resonance frequency and connected to the imaging system via a coaxial cable. This combination allows both transmission and reception of the signals. By manipulating the mutual inductance between the coils, tuning and matching properties of the probe could be adjusted in an effort to achieve optimized power transfer efficiency. A fine-tuning in frequency is achieved by mechanically bending the SC while impedance matching was achieved by adjusting the distance between the PC and SC after they were aligned for proper coupling [4]. Unfortunately, using this strategy, it is not always possible to optimize the mutual coupling, especially when there is a physical constraint preventing the alignment of the coils or adjustment of the distance between the coils. In other cases, the tissue environment surrounding the implanted coil goes through changes that alter the frequency response characteristics of the coil system. This can lead to degraded imaging performance, particularly in the later part of the longitudinal studies. Once implanted, the SC cannot be accessed physically, and therefore its resonance frequency can no longer be re-tuned with our previous coil setup.

In this paper, we provide a practical solution to a real problem that we face in our experimental SCI studies. Specifically we demonstrate herein that the tuning and matching properties of the implanted coil can be modified as needed by inductively overcoupling this coil to an external volume coil with tuning and matching capability. In the following, we provide the theoretical basis for such an approach and demonstrate how it can successfully be utilized in practice using an implantable coil as the SC and a standard capacitively-tuned birdcage volume coil serving as the PC. We provide images from phantom studies and *in vivo *animal studies to demonstrate the reliability and repeatability of the method. Discussion points include explication of the benefits, tradeoffs and limitations that this strategy yields, all within the context of maintaining the image quality in longitudinal studies.

## Theory

**Figure 1:**
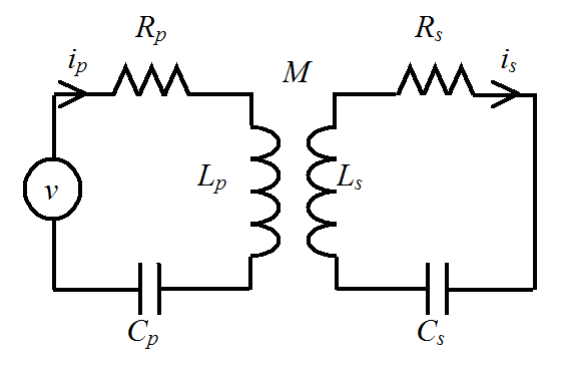
Equivalent circuit diagram of a radio frequency probe consisting of two inductively coupled coils. Circuits in each coil consist of resistive *R*, capacitive *C *and inductive *L *elements. The subscripts *p *and *s *denote primary and secondary, respectively. The mutual inductance *M *between the coils is related to the coefficient of coupling *k *via the formula . *v *denotes voltage source.





The simplified electrical circuit in Fig. 1 is typically used to represent two coils coupled inductively using equivalent lumped-elements [1, 2, 13]. Each coil in the figure consists of resistive *R*, inductive *L *and capacitive *C *elements connected in series. The subscripts *p *and *s *denote primary and secondary, respectively. The mutual inductance *M *between the coils is defined by the coefficient of coupling *k *and the inductive elements according to the formula . Each coil is a self-resonant series circuit tuned at an angular frequency  and described by quality factor *Q*_*i *_= ω*_oi_L_i_/R_i _*for *i *= *p *or *s*. The PC is connected to a voltage source *v*. After applying circuit analysis, the PC and SC currents *i*_*p *_and *i*_*s *_in this arrangement can be written as a function of frequency *f *via the relation ω = 2π*f *as

where *j *is an imaginary unit. To analyze the frequency response characteristics of these currents and the resulting magnetic fields during transmission, we consider, without loss of generality, that the coil system, i.e., the combined coil set, is to be used for operations at 400 MHz, corresponding to the magnetic field strength of 9.4 T, the SC is loaded and |*v*| = 1. Also, we scale the currents and conveniently study *I*_*p *_= *L*_*p*_*i*_*p *_and  (both have unit of AmpereXHenry) as a function of the parameters *k*, ω, *Q*_*i *_and ω_0*i *_for *i *= *p *or *s*.

### Frequency response analysis of the coil system

#### Coil system with f_0*p*_>> f_0*s*_

**Figure 2:**
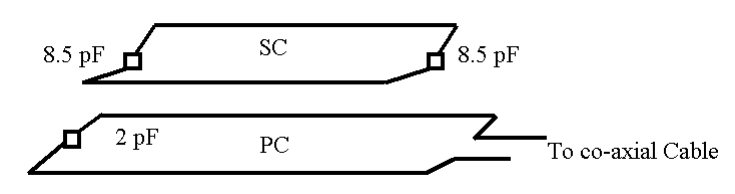
Diagram of an inductively-coupled coil system used in our imaging studies at 9.4 T magnetic field strength. The coil at the top is the SC formed with two 8.5 pF ceramic chip capacitors soldered to two segments of 18 gauge wires to form a pitched rooftop-shaped rectangular loop with dimensions 20 mm × 15 mm. The PC at the bottom is rectangular in shape with dimensions 25 mm × 40 mm and has a 2 pF ceramic chip capacitor attached to one end and the other end is attached to the scanner system via a co-axial cable.

**Figure 3:**
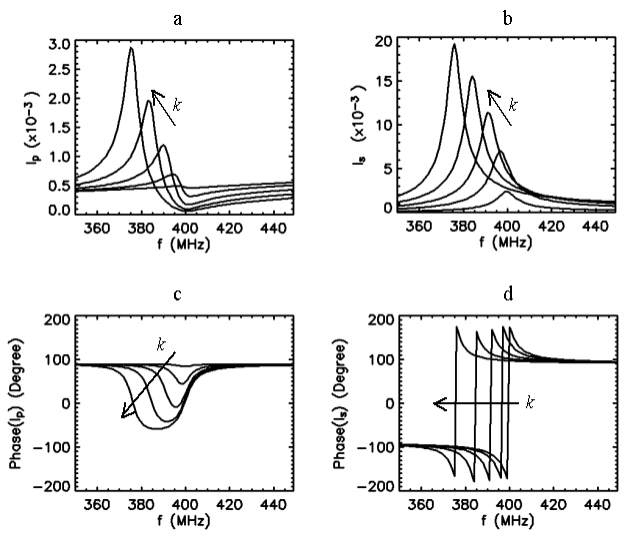
Frequency response curves (amplitude and phase) of the primary (a,c) and secondary (b,d) coil currents for five different values of coupling coefficient *k *= 0.1,0.3,0.5,0.7 and 0.9, when *Q*_*p *_= *Q*_*s *_= 50, *f*_0*s *_= 400 MHz and *f*_0*p *_= 1000 MHz. Arrows indicate the direction of increased k values.

We first analyze the coil system when the PC and SC are tuned individually to a different self-resonant frequency, but with *f*_0*p*_*>> f*_0*s*_. The system shown in Fig. 2 falls under this condition. This setup, where both coils are formed by fixed capacitive and inductive elements, has been used in our imaging studies. The SC is tuned to *f*_0*s *_= 400 MHz, but the PC is tuned to a much higher off-resonance frequency. By assuming that both coils have the same quality factor, the frequency response of the currents *I*_*p *_and *I*_*s *_are plotted for frequencies between 350 and 450 MHz in Fig. 3 for five different coupling values. For weak coupling *k *= 0.1, current is induced in the SC and the response curve of this current peaks at its self-resonance frequency of 400 MHz. When the coupling is increased the position of this peak shifts towards lower frequencies with increasing amplitude. The PC curves exhibit peaks that are aligned with that of the SC curves. The phase difference between the PC and SC currents is initially about 90° at the peak frequency of the SC current and increases with coupling since the unwrapped phase of the SC current increases while the phase of the PC current descreases. The coil system becomes purely resistive at frequencies where the phase of the PC current attains zero. According to the phase data presented in Fig. 3 for the PC, not every coupling produces purely resistive input impedance. If the degree of coupling is high enough, then the system becomes resistive at two different frequencies where the phase of the PC current attains zero.

In practice, we desire adequate coupling to optimally match the impedance. Under the conditions of strong coupling, the curves in Fig. 3 indicate that the current induced in the SC attains higher values than that of the PC. Since currents in both coils collectively determine the pattern of the excitation field (B_1_), the coil with larger current dominates this pattern. When *Q*_*s *_is improved, the PC and SC current response curves can be shown to become sharper, while the behaviors described above remain unchanged.

Combining these observations, the sensitive region of the probe, as defined by the tissue excitation profile and signal reception, under the arrangement in Fig. 2 can be seen as mostly determined by the features of the SC. Typically, the SC is tuned to a higher frequency to start with, so that the combined coil system would yield the SC to resonate at 400 MHz after the impedance matching [4]. When the SC is implanted, it cannot be accessed physically unless a new surgery is performed. This makes it impractical to correct any deviation from the desired resonance frequency if the same PC in Fig. 2 is used as the external coil. However, coupling the implanted coil to an external coil with tuning and matching circuitry can offer retuning ability for the coil system, as explained below.

#### Coil system with f_0*p *_= f_0*s*_

**Figure 4:**
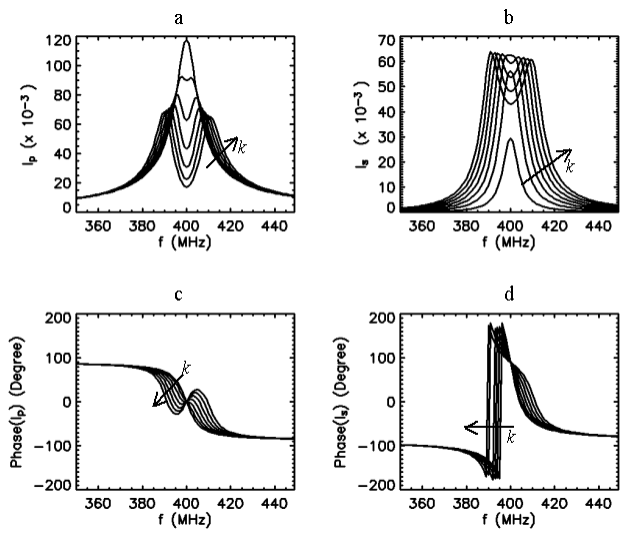
Frequency response curves of the primary (a,c) and secondary (b,d) currents for 7 different values of coupling coefficient *k *= 0.005, 0.0125, 0.02, 0.0275, 0.035, 0.0425 and 0.05 when *Q*_*p *_= 50, *Q*_*s *_= 50, and *f*_0*s *_= *f*_0*p *_= 400 MHz. Arrows indicate the direction of increased k values.



Next, we consider when both the PC and the SC are tuned to same resonance, i.e., *f*_0*p*_* = f*_0*s *_= 400 MHz and possess the same quality factor *Q*_*p *_= *Q*_*s *_= 50. The frequency response characteristics of the probe for this case have been described in detail [13]. Under this configuration, the resulting response curves for the currents in the coupled coils depend very largely on the coupling, as shown in Fig. 4. When coupling is weak *k*~0, using Eq. (1), we can show that *I*_*p *_asymptotically approaches to . This indicates that the response of the PC current approximates the series resonance curve of the primary circuit considered alone. But, from Eq. (2), *I*_*s *_approaches to

indicating that the response curve of the SC current is governed by a shape approximating the product of the resonance curves of the PC and SC circuits taken alone. Other important noticeable features of the graphs in Fig. 4 are that as the coupling is increased, the curve of the PC current becomes broader and its peak value is reduced. At the same time, the curve of the SC current becomes larger and its sharpness is reduced. These trends continue until the coupling reaches a critical value where the peak of the SC current reaches a maximum. By this time, the peak of the PC current has already separated into two peaks. Beyond this critical coupling, the peak of the SC splits. With greater coupling, the double humps on the curves of both PC and SC currents separate farther apart, but their amplitudes and spread remain nearly the same. Under the condition of overcoupling (for our purposes, we define overcoupling as the condition beyond the break point where the SC current peak splits), this behavior in the peaks still remain unchanged, even when the quality factors of the coils are different, i.e., *Q*_*p*_≠*Q*_*s*_.

**Figure 5:**
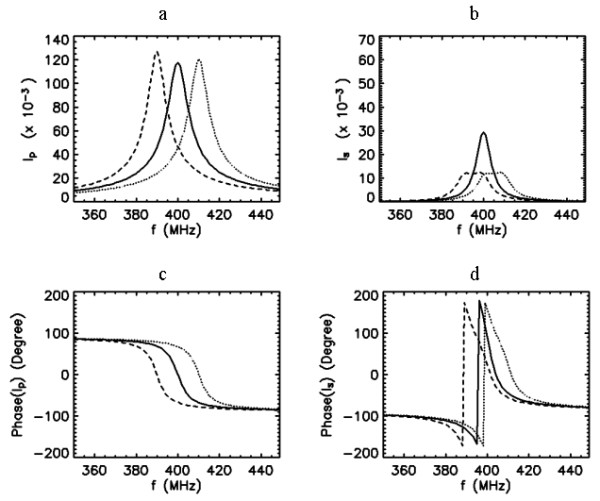
Frequency response curves of the primary (a,c) and secondary (b,d) currents under the weak coupling *k *= 0.005 while *Q*_*p *_= 50, *Q*_*s *_= 50, and *f*_0*s *_= 400 MHz and *f*_0*p *_= 390 (dashed line), 400 (solid line) and 410 (dotted line) MHz.

Under the condition of weak coupling, it is possible to shift the resonance peak of the SC by manipulating the tuning properties of the PC, as shown in Fig. 5. However, this reduces the magnitude of the SC current (and hence the B_1 _field that it produces) while broadening its peak. For these reasons, weak coupling may not be the preferred choice for tuning this coil system. Nevertheless, Hoult and Tomanek [1] considered two circular coils (one for matching and the other acting as a surface coil) arranged coplanar, and their asymptotical analysis on the coil currents predicted 90° phase difference between the currents. According to curves in Figs. 5-c and 5-d, the phases of the primary and secondary currents at 400 MHz respectively read 0° and 90° (with difference of 90°) under the condition of weak coupling and the coils tuned to the same resonance. This phase behavior agrees with the prediction by Hoult and Tomanek [1]. The magnetic flux lines produced by these currents also exhibit quadrature phase relationship at resonance [5]. When the flux lines are not parallel geometrically, elliptically polarized, instead of circularly polarized, field with spatially varying properties is created. This ultimately leads to asymmetry in the excitation field [1].

Strong coupling between the coil components is, in general, an undesired feature in coil design, and is should be avoided whenever possible, as in the case of array coils for parallel imaging. However, in our application, peak-splitting resulting from strong coupling offer an opportunity to externally tune the probe when the SC contains fixed circuit elements. As shown below, either of the two new peaks after the split can be employed for this purpose. One possibility is to initially tune the PC and SC to a frequency higher than the operation frequency of 400 MHz independently, i.e. while the coils are uncoupled *k *= 0. Coupling the coils split the peak of the SC. By manipulating the degree of coupling, the location of the first hump in the frequency axis can be tuned to 400 MHz. If the second hump is desired for the operation, the coils are initially tuned to lower frequency to start with, and then this hump in the SC current is tuned to 400 MHz by changing the coupling strength. Although, these two are equally valid approaches that grant tuning ability to the combined coil system, they still lack taking full advantage of the matching and tuning abilities that may be available in the PC.

#### Coil system with f_0*p *_≠ f_0*s*_

So far, we have considered only the case where both the PC and the SC are tuned to the same resonance frequency. We now consider cases where the PC is tuned at frequencies different than the SC, and demonstrate how this provides additional tuning ability for the combined coils.

##### Resonance at the first peak after the split

**Figure 6:**
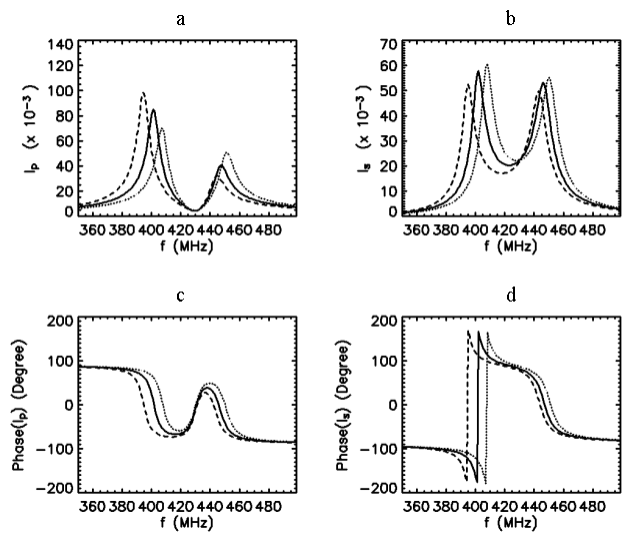
Frequency response curves of the primary (a,c) and secondary (b,d) currents when *k *= 0.1, *Q*_*p *_= 50, *Q*_*s *_= 50, and *f*_0*s *_= 430 MHz and *f*_0*p *_= 405 (dashed line), 415 (solid line) and 425 (dotted line) MHz.

**Figure 7:**
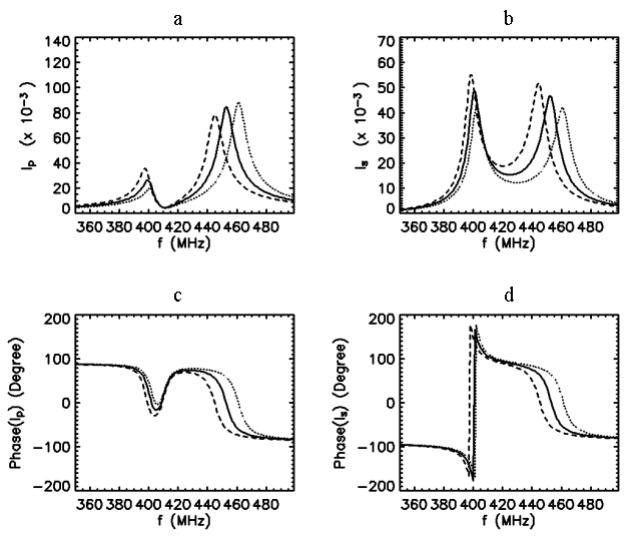
Frequency response curves of the primary (a,c) and secondary (b,d) currents when *k *= 0.1, *Q*_*p *_= 50, *Q*_*s *_= 50, and *f*_0*s *_= 410 MHz and *f*_0*p *_= 430 (dashed line), 440 (solid line) and 450 (dotted line) MHz.

First, we select the first peak after the split to be the resonance peak, and would like to shift the position of this peak by manipulating *f*_0*p*_, which can easily be accomplished using the tuning and matching scheme available in the PC. This goal can be achieved under two conditions on the free-ringing properties of the coils: *f*_0*s*_>*f*_0*p *_or *f *_0*s*_<*f*_0*p*_. As shown in Fig. 6, for the case *f*_0*s*_>*f*_0*p*_, coupling the SC tuned independently to *f*_0*s *_= 430 MHz to the PC tuned independently to *f*_0*p *_= 415 results in the first peak centered at 400 MHz. Changing the tuning properties of the PC from 405 to 425 MHz provide a tuning range for the first peak of the SC between the frequencies 390 and 410 MHz. Or as in Fig. 7, representing the case *f*_0*s*_<*f*_0*p*_, coupling the SC tuned to *f*_0*s *_= 410 MHz to the PC tuned to *f*_0*p *_= 440 MHz again centers the first peak at 400 MHz. Retuning the PC from 430 to 450 MHz shifts the first peak of the SC between the frequencies 395 and 405 MHz. Comparing the curves in Figs. 6 and 7, the arrangement with *f*_0*s*_>*f*_0*p *_can be seen as providing increased tuning range of 20 MHz as compared to 10 MHz achieved with the arrangement *f*_0*s*_<*f*_0*p*_. On the other hand, the current in the PC with the former arrangement reads about four times greater (~0.08 versus 0.02) while the SC current is about 50. This difference in the PC current may have significant implications in the imaging performance. It is also important to note that the phase differences between the PC and SC currents around 400 MHz in Figs. 6 and 7 are about 180°, i.e. out of phase, implying that the flux lines produced by these coils are opposing. Therefore, choosing the first peak as resonance yield the coils to produce B_1 _fields that are destructive.

##### Resonance at the second peak after the split

**Figure 8:**
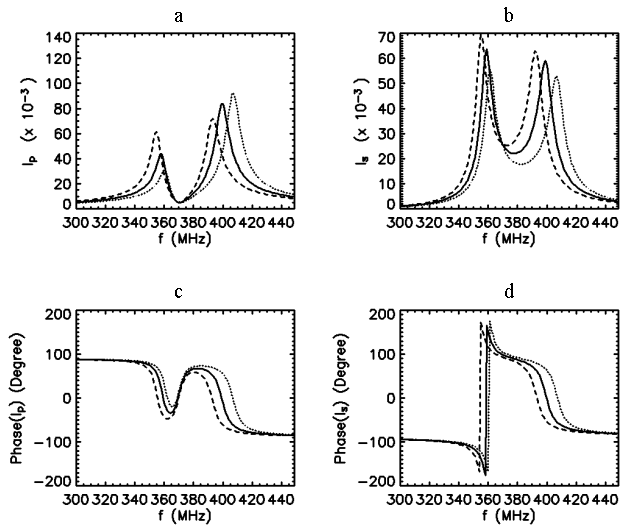
Frequency response curves of the primary (a,c) and secondary (b,c) currents when *k *= 0.1, *Q*_*p *_= 50, *Q*_*s *_= 50, and *f*_0*s *_= 370 MHz and *f*_0*p *_= 375 (dashed line), 385 (solid line) and 395 (dotted line) MHz.

**Figure 9:**
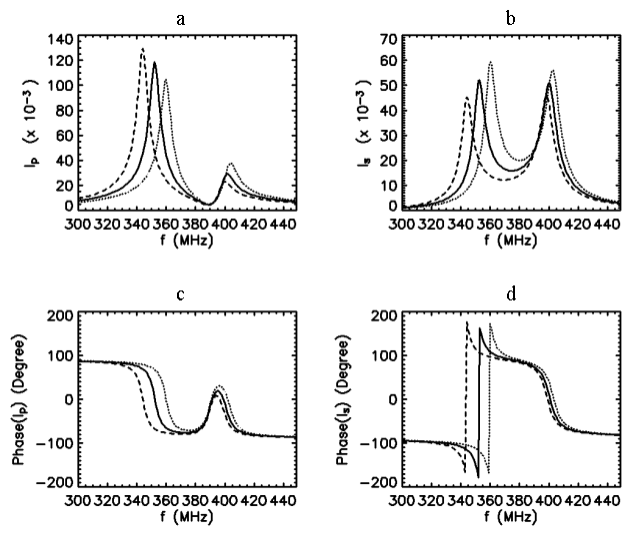
Frequency response curves of the primary (a,c) and secondary (b,d) currents when *k *= 0.1, *Q*_*p *_= 50, *Q*_*s *_= 50, and *f*_0*s *_= 390 MHz and *f*_0*p *_= 350 (dashed line), 360 (solid line) and 370 (dotted line) MHz.

If the second peak is selected as the resonance peak of the combined coil system, manipulating *f*_0*p*_, using approaches similar to those described above, can also shift the position of this peak. Figure 8 demonstrates the second peak tuning between 390 and 410 MHz when *f*_0*p *_is varied from 375 to 395 MHz while *f*_0*s *_= 370 MHz <*f*_0*p*_. Figure 9 shows that the second peak tunes between 395 and 405 MHz when *f*_0*p *_is varied from 350 to 370 MHz while *f*_0*s *_= 390 MHz >*f*_0*p*_. The former approach in this arrangement provides a greater tuning range, but it results in about 4-fold increase in the PC current. From Figs. 8 and 9, the PC and SC currents around 400 MHz can be seen to be in phase (phase difference ~0°) implying that the B_1 _fields produced by these currents are in the same direction and therefore constructive.

From the information provided within the theoretical computations and analysis, the tuning and matching enterprise of the inductively coupled coils can be seen as diverse in nature. In the following, we select one of the overcoupled configurations analyzed in Figs 6, 7, 8 and 9 and apply it into practice to image the rat spinal cord *in vivo*. The tuning and matching range available in our volume coil was the main criterion on the selection of the specific coil configuration. This resulted in the coil configuration with the frequency response characteristics described in Fig. 8.

## Methods

### Components of the coil system

**Figure 10:**
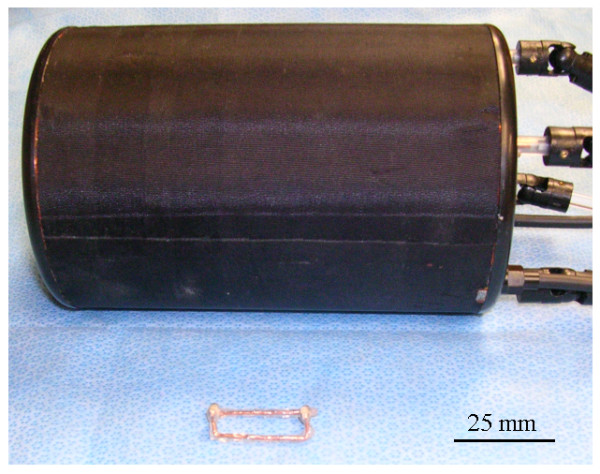
Elastomer coated implantable coil (in the front) and standard volume coil (in the back) used in these studies. Note that only one channel of the quadrature volume coil is used for imaging and the other channel was terminated.

The implantable SC and the primary volume coil used in the studies are shown in Fig. 10. The implantable coil was built from two 8.5 pF ceramic chip capacitors (American Technical Ceramics, Huntington Station, NY) soldered to two pieces of circular wire (18 gauge, CDA 102) (MWS Wire Industries, Westlate Village, CA) to form a rooftop-shaped rectangular loop with dimensions 25 mm × 13 mm. The SC was coated with biologically inert silicone elastomer MDX4-4210 (Factor II, Inc., Lakeside, AZ) for electrical isolation from its surroundings. The PC was a 6 cm inner diameter high pass quadrature birdcage coil with 16 elements, provided by the manufacturer of the scanner. We used only one channel of this coil and terminated the other channel with a 50 ohm resistor.

It is important to distinguish this coil setup from those employed in previous studies where a matching surface coil with tuning and matching circuitry was used as the PC and coupled to a volume coil with fixed elements serving as the SC [2]. In our application, the roles and configurations of the coils are reversed. Also, in previous studies, stent and wireless coils were used to amplify the excitation field generated by large body coil during transmission and couple the signal detected from the resulting magnetization to a surface coil during the receiving phase [5,6]. In contrast to these coil setups, our coil system is simpler in design since the PC is used for both transmit and receive, and does not require complicated electronics required for switching or detuning purposes.

### Coil implantation

The SC was implanted subcutaneously in a Sprague Dawley rat adjacent to the spinal cord at the thoracic level by following the procedures described earlier [4,9]. The rat was maintained under isoflurane anesthesia delivered through a nose mask and monitored using an MR-compatible small animal monitoring and gating system (Model 1025, SA Instruments, Inc., Stony Brook, NY). This system was also used for respiratory-gated acquisition to minimize the breathing-related image artifacts. The temperature of the rat was kept at 37°C by circulating warm air with 40 % humidity using a 5 cm diameter plastic tubing fitted at the back door of the magnet bore.

### Imaging

The resonance frequency of the implanted SC was measured using an external rectangular loop (similar to the one depicted in Fig. 2) attached to a frequency sweeper (Morris Instruments, Inc., Ottawa, Ontario, Canada). For imaging, the rat was placed supine on a Plexiglas tube that was cut half along the long axis, and the tube was inserted into the volume coil such that the implanted coil stayed at its center. Next, to improve the coupling, the volume coil was rotated slightly with respect to the tube until two peaks appeared near the proton resonant frequency of 400 MHz on the sweeper's display. The tuning and matching rods of the volume coil were then engaged to further improve the impedance matching and frequency tuning properties of the second peak observed on the display.

MRI was performed on a 9.4 T horizontal bore scanner (Varian Inc., Palo Alto, CA) using 12 cm ID gradient coil. The Plexiglas sled, supporting the animal and the volume coil, was inserted into the scanner bore. Scout images were first acquired to confirm the placement of the rat at the magnet isocenter. Next, the transmit power of the volume coil was optimized using standard spin echo (SE) sequence to position the 90° excitation band on the spinal cord. Gradient echo (GE) and SE sequences were then employed to demonstrate the excitation field of the combined volume and implanted coils in large field-of-view (FOV) selected in axial, coronal and sagittal planes. The acquisition parameters for the SE data were *T*_R_/*T*_E _= 2500 ms/10 ms, image matrix = 128 × 256, slice thickness = 1 mm and NEX = 2, FOV = 45 mm × 45 mm for the axial, 35 mm × 85 mm for the coronal and 45 mm × 85 mm for the sagittal views. The parameters for the GE data were *T*_R_/*T*_E _= 40/3 ms, flip angle = 45°, image matrix = 128 × 128, slice thickness = 2 mm and NEX = 2, FOV = 45 mm × 45 mm for the axial, 35 mm × 85 mm for the coronal and 45 mm × 85 mm for the sagittal views. Also, high-resolution SE images were acquired in the same planes but in smaller FOV using *T*_R_/*T*_E _= 2500 ms/10 ms, image matrix = 128 × 256, slice thickness = 1 mm and NEX = 2, FOV = 15 mm × 20 mm for the axial, 24 mm × 33 mm for the coronal and 15 mm × 33 mm for the sagittal views. These scans were repeated on days 7 and 14 of the coil implantation to show the repeatability of the scans at the same level of imaging quality. These studies were performed under a protocol approved by the institutional animal care and use committee at the University of Kansas Medical Center. Additional scans were performed on a uniform phantom, consisting of a 15 cc plastic tube filled with 0.9% NaCl water solution, by placing the SC on the surface of the tube.

## Results and Discussion

The implantable SC exhibited self-resonance at 407 MHz in bench tests in air (i.e., unloaded), measured with the coil configuration shown in Fig. 2. The resonance peak was sharp with quality factor of about 150. When implanted into the rat, the resonance frequency of the loaded coil shifted down to 388 MHz and its quality factor dropped to about 30. The volume coil was initially tuned to 400 MHz when it is unloaded. After inserting the rat into the volume coil, a single peak was observed on the frequency response curve, which indicated weak coupling between the implantable SC and the volume coil. Rotating the volume coil with respect to the rat increased the coupling and produced double peaks. The second peak was then tuned and matched at 400 MHz by varying the matching and tuning capacitors of the volume coil. At this time, the first peak was positioned at 379 MHz, was broader and had lower amplitude as compared to the second peak. These resonance properties closely resembled the features of the theoretical case presented in Fig. 8. After placing the rat into the scanner's bore, the peaks on the response curve shifted about 1 MHz to lower frequencies, and were readjusted again finely.

For imaging the spinal cord properly with the setup described above, the transmit power was optimized at 23 dB when the 90° rf pulse was 2 ms long. This power level was comparable to the power used when the rectangular loop in Fig. 2 was employed as the external coil. If the volume coil were used alone in quadrature mode to image the rat body, the optimal power for the same pulse would typically be achieved with much higher 42 dB power in our scanner.

**Figure 11:**
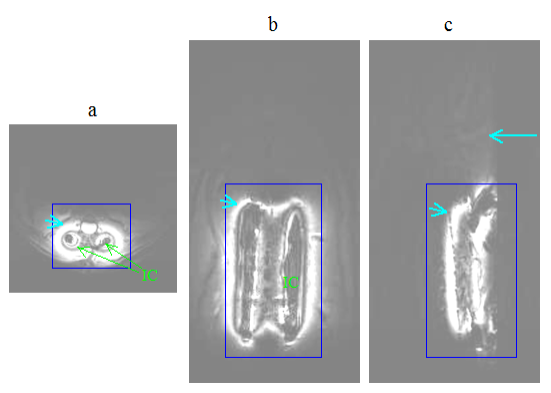
*In vivo *spin-echo images of spinal cord in (a) axial (b) coronal and (c) sagittal planes acquired on the day of coil implantation using the parameters *T*_R_/*T*_E _= 2500 ms/10 ms, image matrix = 128 × 256, slice thickness = 1 mm and NEX = 2, FOV = 45 mm × 45 mm for the axial, 35 mm × 85 mm for the coronal and 45 mm × 85 mm for the sagittal views. The intensities in the images were windowed and scaled to enhance the background signal. Notice that, during transmission, the volume coil generates an excitation field that leads to the hypointensity (long arrow) seen in the background. The implanted coil, indicated by IC on the images, generates 90° excitation field that produces a band of hyperintensity (short arrows) surrounding it. The square boxes are used to localize the regions where the images in Fig. 14 below were acquired.

**Figure 12:**
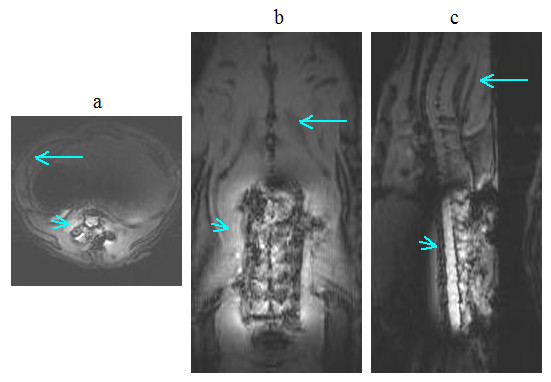
*In vivo *gradient echo images of spinal cord in (a) axial, (b) coronal and (c) sagittal planes acquired on the day of coil implantation using the parameters *T*_R_/*T*_E _= 40 ms/3 ms, flip angle = 45°, image matrix = 128 × 128, slice thickness = 2 mm and NEX = 2, FOV = 45 mm × 45 mm for the axial, 35 mm × 85 mm for the coronal and 45 mm × 85 mm for the sagittal views. The intensities in the images were windowed and scaled to enhance the background signal. Notice that, during transmission, the volume coil generates an excitation field that leads to the hypointensity (long arrows) seen in the background. The implanted coil generates excitation field that produces the hyperintense footprint (short arrows).

**Figure 13:**
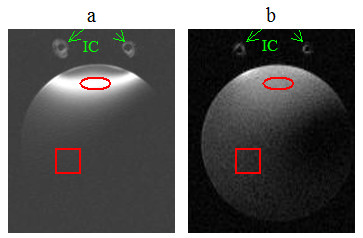
Axial spin-echo (a) and gradient-echo (b) images of a 15 cc tube filled with 0.9% NaCl water solution. IC denotes implantable coil. The circles and squares respectively denote the regions of interest selected to measure the mean signal intensities in the excitation field of the implanted coil and the background regions excited by volume coil. The intensities in the images were windowed and scaled to enhance the background signal.

As discussed in the previous section, the currents in both coils were expected to produce excitation fields in the rat's body during transmission. The images in Figs. 11 and 12, obtained specifically with large FOV, delineate tissue not only in the footprint of the implanted coil but also in the background regions. This behavior can better be understood from the images shown in Fig. 13, which were produced during studies with the uniform phantom. The homogeneous weak signal in the background is induced as a result of the excitation by the volume coil. But, the inhomogeneous field seen near the site of the implanted coil is formed by contributions from both coils, but mainly by the implanted coil. We selected two regions of interest; one within the 90° field of the implanted coil and the other in the background field produced by the volume coil, as indicated in Fig. 13, and measured the mean signal levels in these zones. The ratios of the means were 18 on the SE image and 6 on the GE image.

**Figure 14:**
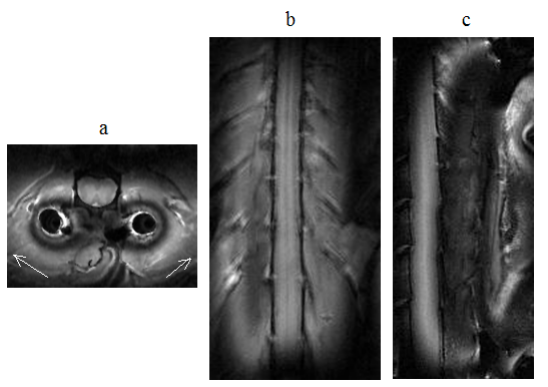
*In vivo *high resolution spin-echo images of spinal cord in (a) axial (b) coronal and (c) sagittal planes acquired on the day of coil implantation using the parameters *T*_R_/*T*_E _= 2500 ms/10 ms, image matrix = 128 × 256, slice thickness = 1 mm and NEX = 2, FOV = 15 mm × 20 mm for the axial, 24 mm × 33 mm for the coronal and 15 mm × 33 mm for the sagittal views. Arrows on the axial image point to wraparound artifacts.

The images in Fig. 14 were acquired with smaller FOV to visualize the anatomy and structure of the rat spinal cord in greater detail. These images clearly demonstrate the capability of the coil system to acquire adequate quality of data to accomplish the high-resolution imaging task while maintaining good contrast between gray matter and white matter. The data inherently contain wraparound signals from the tissue excited by the volume coil outside the selected FOV. As discussed above, these signals are significantly low in intensity when the SE acquisition is employed, and subsequently produce negligible artifacts. But, wraparound artifacts produced by the signals from the tissue near the implanted coil remain strong and are visible on the images as fold over. If the FOV is selected large enough, these artifacts move towards the edges, allowing the spinal cord be visualized at the center of the image in a nearly artifact free fashion.

**Figure 15:**
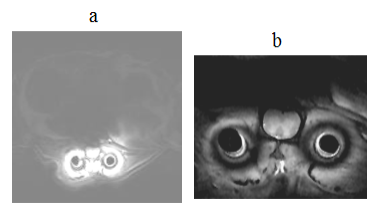
*In vivo *axial spin-echo images of spinal cord acquired on day 14 of the coil implantation in (a) larger view of 45 mm × 45 mm and (b) smaller view of 15 mm × 20 mm using the parameters *T*_R_/*T*_E _= 2500 ms/10 ms, image matrix = 128 × 256, slice thickness = 1 mm and NEX = 2. The image intensity in (a) was windowed and scaled to enhance the background signal.

On days 7 and 14 of the coil implantation, the self-resonance peak of the implanted coil respectively read 385 MHz and 381 MHz, which were lower than 388 MHz measured on the day of implantation. When the rat was placed in the volume coil outside the magnet, double peaks were observed. The second peak was tuned and matched at 400 MHz for imaging. Figure 15 shows axial images of the spinal cord on day 14, acquired with the same parameter values used to produce the images in Figs. 11-a and 14-a. Qualitatively, the axial images acquired on both days can be seen as comparable, demonstrating the capability of the coil system to provide consistent data from the same subject scanned two weeks apart even when the tuning frequency of the implanted coil was shifted. If such frequency shift were to occur while using the coil setup in Fig. 2, the imaging performance would be compromised.

Besides local field uniformity and high SNR, performance, practicality, reliability and repeatability are the key properties of the rf probes, that are highly-desired in longitudinal studies. Using the above coil system, the implantable coil produces a narrow strip of 90° excitation field that is sufficiently wide enough to uniformly image the spinal cord. The small footprint of the excitation allows high resolution imaging of the local tissue. But the resulting images inherently contain wraparound artifacts from the field generated by the volume coil. The sequence and the parameters used for imaging influence the severity of these artifacts. Our data from Fig. 13 indicate that the SE sequence produces relatively little artifacts while maintaining the sensitivity and specificity of the coil intact. A less obvious drawback of the coil system is that the large size volume coil produces more noise in the received signal as compared to a small pickup coil. Also, the effective bandwidth of the coil system increases with overcoupling. Noise (produced externally or thermally) that would be filtered otherwise can therefore leak though the frequency band at the second peak not used for the operation. However, the benefits of the overcoupled coil system overweigh its shortcomings.

In conclusion, the results from the systemic investigations presented above demonstrate that the system satisfies the properties expected from rf coils. The system performs well in acquiring high quality *in vivo *MR data longitudinally. Readily available standard volume coils enhance the system by providing tuning and matching capabilities. This provides practicality by minimizing the time spent in preparing the animal for the scans, reliability and repeatability by ensuring that the data will be acquired continuously in longitudinal studies. In other aspects, additional benefits of using this coil system include simplicity, low-cost and flexibility. It does not require active/passive detuning of the SC during transmission or coil combinations that involve coupling the SC to a surface coil or a like during reception [3,5,6]. Also, manufacturing the implantable coil in different shapes and sizes by properly cutting and forming the wires and soldering the capacitors allows local imaging of internal structures when the coil is implanted or placed on the surface. Given the benefits of this type of coil system, it is likely to prove to be highly useful in gathering longitudinal MR imaging data in experimental studies.
